# A Case Study of a Deaf Autistic Adolescent’s Affective and Linguistic Expressions

**DOI:** 10.3390/bs15111435

**Published:** 2025-10-22

**Authors:** Kristin Walker, Jenny L. Singleton, Aaron Shield

**Affiliations:** 1 Department of Psychology, Stony Brook University, Stony Brook, NY 11794, USA; kristin.walker@stonybrook.edu; 2 Department of Linguistics, Stony Brook University, Stony Brook, NY 11794, USA; 3 Speech Pathology & Audiology, Miami University, Oxford, OH 45056, USA; shielda@miamioh.edu

**Keywords:** deaf, autistic, sign language, nonmanual markers, facial expressions

## Abstract

Facial expressions and body language play crucial roles in communication by conveying emotional and contextual information. In signed languages, facial expressions also serve linguistic functions. While previous research on autistic individuals’ facial expressions has focused primarily on affective expressions in hearing people, studying deaf autistic individuals offers insight into how autism affects linguistic and affective facial expressions. This case study examines the nonmanual expressions of “Brent,” a Deaf autistic adolescent natively exposed to American Sign Language (ASL). Five video recordings (four monologues and one conversation, totaling 35 m) were coded for nonmanual expressions, including affective facial expressions, question marking, negation, and other functions. Across 590 coded utterances, Brent showed absent or reduced facial expressions for both linguistic and affective purposes. However, he frequently used alternative communicative strategies, including additional manual signs, sign modification, and body enactment. Use of body movement to convey negation, affirmation, or emphasis was observed but inconsistently applied. These findings expand the current understanding of how autistic individuals use facial expressions by including linguistic functions in a signed language and support a broader view of autistic communication that embraces diverse and effective languaging strategies beyond neurotypical norms.

## 1. Introduction

### 1.1. Author’s Positionality Statement

In this case study of “Brent,” a Deaf autistic adolescent, we use identity-first language, aligning with the preferences of many in both the autistic (Bottema-Beutel et al., 2021) and Deaf (American Psychological Association [APA], 2023) communities. We capitalize “Deaf” to indicate Brent’s cultural and linguistic identity as a member of the Deaf community and a natively exposed signer with Deaf family members.^1^ Importantly, all three authors transparently share our positionality as nonautistic, hearing individuals, recognizing that our identities may shape our perspectives. Author 2 (J. Singleton) is a hearing natively exposed signer of American Sign Language (ASL), while Authors 1 (K. Walker) and 3 (A. Shield) are hearing, late learners of ASL and proficient signers. In terms of professional expertise, the authors are a doctoral candidate in clinical psychology and two professors of Linguistics, who specialize in signed languages, autism, and language patterns of disabled signers.

We adopt the term *languaging* to describe Brent’s dynamic process of meaning-making, which includes his use of signs, gestures, facial expressions, and body language to communicate. Furthermore, instead of solely focusing on how Brent’s communication differs from normative expectations, we highlight his unique, creative ways of communicating. This strength-based approach places value on the richness and diversity of languaging among deaf autistic individuals (see Henner & Robinson, 2023, for a relevant discussion of languaging and Crip Linguistics).

### 1.2. Background

Autism is notably more prevalent within the deaf and hard-of-hearing population than in the general population, with estimates ranging from 1.9% (Szymanski et al., 2012) to 5.8% (Kancherla et al., 2013), compared to approximately 2.8% in the general population (Maenner et al., 2023). Despite this elevated prevalence, there remains a limited body of research focused specifically on deaf autistic individuals, especially those who are natively exposed to signed language from birth.

Over the past decade, several studies have begun to document the signing patterns of deaf autistic children raised in ASL-using households. These studies have reported, for example, palm reversals (Shield & Meier, 2012; Shield et al., 2020), imitation differences (Shield & Meier, 2018), pronoun avoidance and the use of names (Shield et al., 2015), motor and articulation challenges (Bhat et al., 2018; Shield et al., 2017b), sign echolalia (Shield et al., 2017a), and perspective-taking and theory of mind difficulties (Shield et al., 2016).

In a prior case study of Brent (Singleton et al., 2023), a Deaf autistic adolescent natively exposed to ASL, we noted strengths and distinctive patterns in his ASL. Among strengths, Brent demonstrated age-appropriate ASL vocabulary, sophisticated semantic content (e.g., he discusses the impact of the COVID-19 pandemic), accurate fingerspelling, and evidence of metalinguistic awareness. Distinctive patterns included phrase repetitions,^2^ a non-standard handshape intrusion, and few affective or linguistic facial expressions. While this prior case study suggested minimal affective and linguistic facial expressions, Brent’s facial expressions were not examined systematically.

This observation is important given the special role that facial expressions play in signed languages. In ASL, “nonmanual markers”—i.e., facial expressions, head movements, and body shifts—encode grammatical/morphosyntactic information (such as negation and questions), prosodic cues, and affective states. Unlike in spoken languages, therefore, in which prosody and affect are considered paralinguistic cues, nonmanual markers in signed languages carry multiple functions, layering linguistic and affective meanings through the use of the face and body.

The multiple functions carried by nonmanual markers in signed languages raise important questions for deaf signers who are autistic. Few studies have examined nonmanual markers in deaf autistic signers. One early case study of an adult Deaf, autistic native signer (“Judith M.”) reported a distinct absence of facial expressions in her signing, suggesting that autism can significantly impact nonmanual expressions even in those with native exposure from birth by their Deaf parents (Poizner et al., 1990).

A different case study reported a hearing autistic adult and polyglot savant, Christopher, who was taught British Sign Language (BSL) in adulthood (Morgan et al., 2002). Although Morgan et al. (2002) did not investigate Christopher’s affective facial expressions specifically, they noted that despite his extraordinary linguistic abilities he did not produce facial expressions that are considered obligatory for marking questions in BSL. However, he did produce nonmanual head shaking to signal negation.

To our knowledge, to date there is just one study that systematically investigated how deaf autistic individuals produce facial expressions. Denmark et al. (2019) analyzed the affective facial expressions produced during a BSL narrative task by nonautistic (*n* = 12) and autistic (*n* = 10) deaf children. Deaf autistic children produced facial expressions that were rated of lower quality than typical deaf children. Autism severity was associated with the production of fewer emotional facial expressions, but linguistic factors (such as narrative quality) did not account for the differences. Denmark et al. concluded that the differences in facial expression production were most likely due to underlying deficits in emotion processing and theory of mind, and that, for these deaf autistic individuals, signed language exposure was not sufficient to overcome such deficits.

Consistent differences have also been found in the emotional expressiveness and prosodic characteristics (intonation, stress, rhythm, and volume of speech) and gestures of some autistic speakers (de Marchena et al., 2019b; Fusaroli et al., 2017). Some hearing autistic individuals display facial expressions less frequently, for shorter durations, and with less perceived clarity or intensity than nonautistic individuals (see Trevisan et al., 2018 for a review). Nonautistic observers often rate autistic individuals’ facial expressions as less accurate or more difficult to interpret, even though some autistic individuals have the physical capacity of producing such expressions.

Therefore, we ask why some autistic individuals’ facial expressions are produced differently than those of nonautistic individuals. Research suggests that alexithymia, or difficulty with identifying and describing one’s emotions, is implicated in the production of facial expressions. Trevisan et al. (2016) found that alexithymic traits, and not autistic traits, were related to the quantity of facial expression production. This “alexithymia hypothesis” argues that emotional processing differences may underlie some of the expressive features typically attributed to autism. While alexithymia is frequently observed in autistic individuals, it is not a universal trait (Kinnaird et al., 2019), indicating a need for further research to disentangle its role. If emotion processing differences underlie the differences in affective facial expressions, it remains an open question if such differences extend to facial expressions that are used in signed languages for linguistic purposes, such as question marking.

### 1.3. Current Study

This study extends our previous analysis of Brent’s ASL use by examining his nonmanual expressions—specifically, his facial expressions and head/body movements—across five video-recorded narratives. Two of these videos were included in our earlier study (Singleton et al., 2023) and are reanalyzed here for nonmanual markers; three additional videos are analyzed for the first time. Notably, one of the new videos features Brent in naturalistic conversation with familiar, non-experimenter partners, offering a novel context for analysis.

We ask:

1. What nonmanual markers does Brent produce across affective, linguistic, and other communicative domains?
2. Do these expressions vary by function (e.g., affective vs. linguistic)?
3. Does Brent utilize any alternate strategies to support his communication in the absence or reduction of conventional nonmanual markers?

We adopt a descriptive, strength-based lens to understand the range and nature of Brent’s nonmanual expressions, including whether any observed differences reflect a global expressive difference, a domain-specific profile, or alternative communicative competencies.

#### Hypotheses

To guide our inquiry, we propose three possible patterns of nonmanual expression in Brent’s signing, based on theoretical considerations and previous findings.^3^ These are summarized in Table 1.

- H0: No Impact (Null Hypothesis)
  Brent will produce both affective/prosodic and linguistic nonmanual expressions in ways consistent with fluent ASL use. This pattern suggests that his native exposure to ASL may act as a protective factor, supporting his development of nonmanual expressions in all domains.
- H1: Global Reduction
  All categories of nonmanual expression (affective/prosodic, linguistic, and other) will be reduced or absent, reflecting a general impact of autism on expressive behavior.
- H2: Affective/Prosodic Domain Selectively Affected
  Brent will show reduced affective/prosodic facial expressions but preserved linguistic nonmanual ASL markers, consistent with the alexithymia hypothesis and previous findings in hearing autistic individuals.

## 2. Method

Five video recordings of Brent, collected across a 3.5 year period (between ages 16;11 and 20;5) were provided by his parents. Informed consent was obtained from Brent’s parents, and Brent himself provided assent to participate. The focus of this study is Brent’s use of nonmanual expressions, and the study received approval or exemption from the authors’ institutional review boards.

### 2.1. Participant

Brent is a natively exposed (acquired signed language from birth) ASL signing Deaf male adolescent on the autism spectrum. He was exposed to ASL from birth from two natively exposed ASL signing hearing parents and four Deaf grandparents. In addition to being exposed to ASL at home, he is, at the start of the videorecordings, in 10th grade at a state residential school for the deaf that provides ASL educational services in their special education program. Brent has received special education services since age five months.

Brent was diagnosed with sensorineural bilateral severe-to-profound deafness (confirmed by age 10 months), autism (diagnosed at age 5), Temple Syndrome (at age 12), and generalized anxiety disorder and major depressive disorder (in partial remission since age 16, before these video recordings take place). Temple Syndrome is a rare genetic condition characterized by various medical issues and distinctive features. Clinical descriptions of Temple Syndrome include several physical traits, such as a broad and high forehead, a small mouth, a triangular face, smaller hands, joint hypermobility, low muscle tone, and potential for clinodactyly (abnormally curved fingers). Based on our subjective observation of Brent, he does appear to have some of these Temple Syndrome physical features. At age 8;3, Brent was evaluated using the Autism Diagnostic Observation Schedule, Second Edition (ADOS-2; Lord et al., 2012), by a research-reliable clinical psychologist fluent in ASL, receiving a total score of 14 on module 2 and an autism severity score of 6, indicating moderate autism. He also obtained a score of 19 on the Social Communication Questionnaire (SCQ; Rutter et al., 2003) parent checklist, above the threshold score of 15 (Chandler et al., 2007). Some of Brent’s autistic traits include focused interests (food, restaurants, stores, and hotels), self-stimulating behaviors, and an insistence on sameness or preference for routines.

In previous work (see Singleton et al., 2023), we characterized Brent’s ASL signing as “fluent,” by which we subjectively mean that he generally engages with interlocutors using lengthy and linguistically complex phrases that are topically relevant, with few communication breakdowns (as evaluated by one of the co-authors who is a native signer of ASL). Objectively, at age 8;5, Brent performed at a level similar to his age-matched native signing peers when he was administered the American Sign Language-Receptive Skills Test (Enns & Herman, 2011; Enns et al., 2013) by another one of the co-authors of this study. We provide further elaboration on Brent’s conversational quality in Section 3.1.

### 2.2. Materials and Procedures

#### 2.2.1. Video Recordings

Five video recordings were analyzed between the ages of 16;11 and 20;5. In four of the recordings (Videos 1, 2, 4, and 5), Brent signs monologue-type narratives for his social media audience, discussing his favorite things and inviting his followers to post their comments. Video 3 features Brent having a conversation with adult family friends. This conversational video was intended to elicit naturalistic data of Brent asking questions, so as to create more opportunities for the production of nonmanual expressions. Most of the conversation is between Brent and a woman, while the end of the conversation shifts to Brent and a man sitting at the same table; both friends are fluent Deaf ASL users.^4^

Brent’s mother, a hearing natively exposed ASL signer and certified signed language interpreter, recorded all of the videos as a passive videographer. She also provided real-time ASL-to-English translation of Brent’s signing. Table 2 presents information about the video recordings, including Brent’s age at the time of recording, the duration of each video, and the primary topic or content of each recording.

#### 2.2.2. Coding

Videos were annotated using ELAN (ELAN, 2024) to create time-aligned annotations. We first transcribed Brent’s mother’s voiced translation. Author 2, a natively exposed ASL signer, reviewed the translation for accuracy and then glossed^5^ each utterance for ASL signs. ASL glosses were reviewed, verified, and confirmed by Author 2 and several undergraduate research assistants.

After the English transcription and ASL glossing were completed, we coded utterances based on contexts in which nonmanual markers are expected, such as questions, negation, affective signs, and other body movements. For each category, we coded for the presence and absence of facial expressions, manual lexical signs, and any other strategies that may convey meaning. We disregarded repeated utterances, counting each meaningful utterance only once. For a more detailed explanation of each category, see Section 2.2.3 below.

As Video 3 captures a conversational context, we added Brent’s nonmanual reactions (e.g., backchanneling) while his conversational partner signed and documented instances of overlapping signing between Brent and his conversational partner. The question status of an utterance was determined by utilizing context (i.e., his communication partner’s response) and facial expressions were noted when Brent’s neutral facial expression changed (i.e., facial expressions were noted when they were considered distinct from the previous utterance’s facial expression).

Two undergraduate research assistants with at least one year of ASL experience coded each video. Author 2 reviewed the two coders’ responses and made iterative modifications to the coding decisions, with the coding categories refined or elaborated through research team discussions between all three authors and the undergraduate research assistants.

#### 2.2.3. Coding Categories

Affective Facial Expressions. Smiling, frowning, scowling, embarrassment, or other facial expressions that are associated with emotion and are used by signers and non-signers alike were coded as affective facial expressions.

Questions. Linguistic analyses of ASL have shown that questions are marked not only through manual signs but also through distinct facial expressions known as nonmanual markers. ASL employs three main types of question marking: Wh- (content) questions, Yes–No (polar) questions, and rhetorical questions.

Wh-Questions. In ASL, it is expected that Wh-questions are marked by furrowed eyebrows over the scope of the utterance. For example:

________________wh

WHERE LIVE YOU = “Where do you live?”

However, a Wh-question can also be expressed without a manual Wh-sign if the furrowed brow is maintained:

__________________wh

MIDDLE NAME YOU = “What is your middle name?”

By contrast, a Wh-question is considered unacceptable if the nonmanual marker is omitted.

The furrowed eyebrow nonmanual marker can also be used without a Wh-manual sign to implicitly pose a WHICH-type question, often via a “choice offering” strategy (Wilbur, 1999):

_____________________wh

SMALL MEDIUM LARGE = “Small, medium, or large? Which one?”

Yes–No Questions. Yes–No (polar) questions are marked by raised eyebrows over the scope of the utterance.

____________y/n

YOU LIKE FISH = “Do you like fish?”

Lexical Tag Questions (LEX-Q). ASL also allows for Yes–No questions to be marked using an explicit manual sign, often a lexicalized sign derived from QUESTION, placed at the beginning or end of a sentence:

________________________y/n

FINISH HOMEWORK LEX-Q = “Did you finish your homework?”

This LEX-Q tag is not a productive or required nonmanual marker for all Yes–No questions. Rather, it tends to be used when the speaker is expressing doubt, emphasis, or urging a response (Valli et al., 2011). It may co-occur with raised eyebrows but does not replace them.

Rhetorical Questions. Baker-Shenk (1985, as cited in Valli et al., 2011) described rhetorical (RhQ) structures in ASL, such as:

   __RhQME TIRED WHY? STUDY ALL-NIGHT = “I am tired…Why?I’ve been studying all night!”

Such embedded rhetorical questions must co-occur with raised eyebrows, even if a Wh-sign is used. Additional cues like a head tilt or slight headshake also may accompany rhetorical questions.

**Figure 1:**
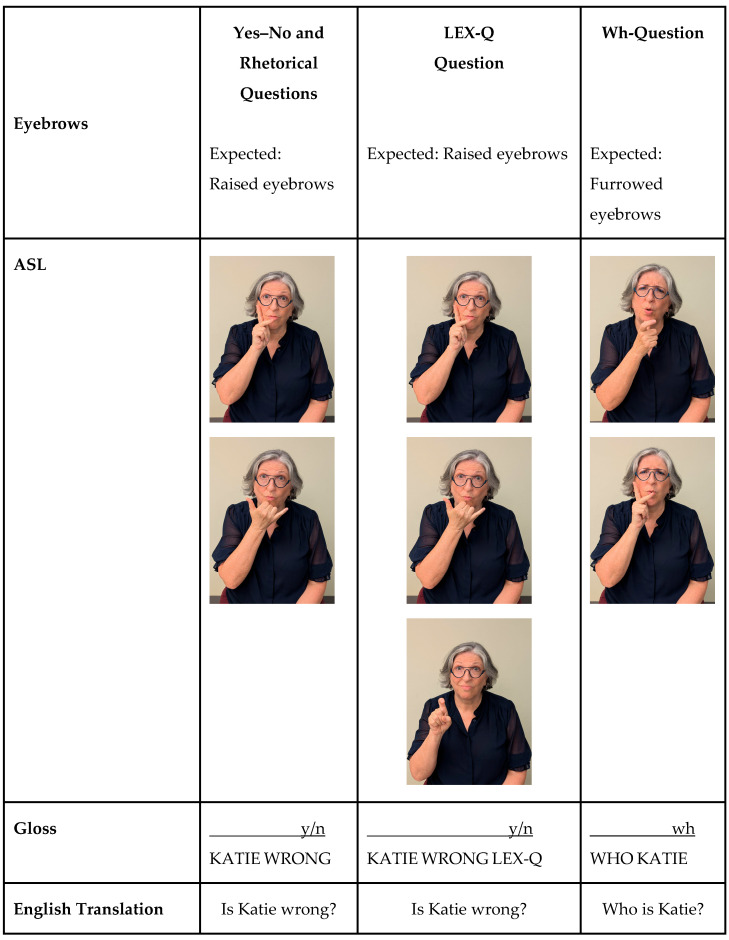
Yes–No and Wh-question marking in ASL. *Note:* ASL glosses use capitalized English words to represent signs. Nonmanual markers (e.g., eyebrow position) are noted above glosses with the length of the line indicating the scope of co-occurring nonmanual marker. Question types are abbreviated (“y/n” = Yes–No and “wh” = Wh-type).

Figure 1 demonstrates how nonmanual markers (e.g., eyebrow position) differentiate Wh- from Yes–No questions.

Negation. We coded instances of negation in ASL using both manual and nonmanual markers. Following prior literature (Veinberg & Wilbur, 1990; Zeshan, 2006), we coded a side-to-side headshake as the primary nonmanual marker of negation. This headshake could occur with or without a manual negator.

Although the linguistic status of the headshake has been debated—some researchers viewing it as syntactic (e.g., Emmorey, 2002; Liddell, 1978, 2003; Neidle, 2000; Pfau & Quer, 2010) and others as prosodic (e.g., Nespor & Sandler, 1999; Atkinson et al., 2004)—we made no theoretical assumptions in our coding. We included any headshake temporally aligned with a clause involving negation, regardless of its grammatical interpretation. Additionally, in line with Gonzalez et al. (2019), we allowed for brow raise (rather than headshake) to mark negative Yes–No questions, coding it as a valid nonmanual marker of negation. For example:

__________y/n 

YOU NEVER? = “Haven’t you ever [done that]?”)

Other Nonmanual Expressions. In addition to nonmanual markers related to questions and negation, we coded a range of other nonmanual behaviors observed in Brent’s signing. These included facial expressions, head movements, and body shifts that, while not necessarily present in expected ASL signing, contribute to meaning or interactional nuance. Because the linguistic status of these behaviors is not firmly established, we documented their presence (rather than absence) to provide a fuller account of Brent’s expressive repertoire.

Head Movements and Body Shifts. We coded four types of head and upper-body movement commonly observed in ASL. Although these behaviors are typically considered prosodic in function (Herrmann, 2013; Wilbur, 2000), they are often meaningfully integrated with manual signs:

1. Affirmative Nod (aff; vertical nodding): Used to reinforce certainty or agreement.
  __________aff
  MIKE GENIUS = “Mike is assuredly a genius”
2. Lateral Head Movement (lhm): Used to convey uncertainty or hesitation
  __________lhm
  MIKE CYCLING = “Mike’s bike-riding is kind of iffy”
3. Backchanneling Nod: A smaller, rhythmic nod used to indicate active listening or encouragement to continue (similar to “mm-hmm, go on”).
4. Coordinate Shift: Alternating head or body shifts used to distinguish referents in a disjunctive construction.

TEA (shift_Loc1_) COFFEE (shift_Loc2_) = “tea or coffee”

     (Coordinate Shift example from Wilbur, 1999).

We also coded emphatic expressions, including combinations of increased facial tension, frowning, head nod or tilt, and sign modification (e.g., changing BLUE to VERY-BLUE via a longer, thrust-like movement). These forms are associated with intensification and adverbial emphasis (Klima & Bellugi, 1979; Sandler & Lillo-Martin, 2006; Wilbur et al., 2012).

Other Motor Behaviors. Given the focus of this study on a Deaf autistic adolescent, we also coded non-signing motor behaviors that are not part of ASL but may be associated with autism or anxiety. These included manual stims (e.g., hand flapping, wringing, or repetitive finger movements), whole-body stims (e.g., pacing, rocking), and facial/head stims (e.g., eye rubbing, head shaking unrelated to linguistic structure). These behaviors were coded in terms of their co-occurrence or interference with ASL-related manual or nonmanual expressions. We paid particular attention to how these behaviors interact with the signing channel—either overlapping with or interrupting communicative expressions.

A summary of all coded nonmanual categories and their associated communicative or affective functions is provided in Table 3. For the full coding scheme, see Appendix A.

#### 2.2.4. Reliability

To assess reliability, two undergraduate research assistants independently coded one video segment (Video 5, December 2024) at the end of the coding period, resulting in 44 comparisons per coding category. Intercoder agreement was calculated using simple proportion of matching codes and was high across all tiers: Repetition (0.93), Question Type (1.00), Negation (1.00), Affect (1.00), and Body Shifts (0.95).

## 3. Results

In total, 590 total utterances were analyzed, excluding repetitions: Video 1 (*n* = 126), Video 2 (*n* = 217), Video 4 (*n* = 43), and Video 5 (*n* = 101). In Video 3, which also involved a communication partner, Brent had 103 conversational turns, each of which had a meaningful utterance (which was often repeated). Additionally, we documented 80 partner turns as well as 10 turns by a third individual (the partner’s husband). Brent was not visible within the frame for 23.7 s of Video 3, so we could not code his nonmanual expressions during that time.

### 3.1. Overall Description of Conversation Quality

We first present an overview of Brent’s communication patterns and overall conversational quality.

Although we did not include repeated utterances in our analysis of nonmanual expressions, repetition of phrases was a dominant pattern across all five videos (between 20% and 94% of utterances in each video were repeated). Most repetitions were twice in number, but a small percentage (2–6%) were higher; see Singleton et al. (2023) for further discussion of Brent’s repetitions.

Video 3 allowed us to examine Brent’s conversational abilities, including communicative actions that support one’s conversational partner and demonstrate engagement, such as initiating conversation, maintaining connected eye gaze, backchanneling, contingent responding, and turn-taking regulation. Brent asked numerous questions (*n* = 34) about his communication partner’s family and life; Brent’s partner asked him 17 questions, two of which went unanswered.

Mutually connected eye gaze was well established between Brent and his conversational partner, as expected in ASL conversations. Brent also successfully shifted his attention to the second conversational partner. Finally, Brent utilized a linguistically appropriate hand wave to obtain the second partner’s attention.

Brent produced backchanneling nods during 22 out of his partner’s 80 conversational turns (27.5%). He wrung his hands during seven of these nods as well as during four additional partner turns without head nodding. This action could be a kind of distinctive backchannel expression, or it may be a kind of self-regulator (i.e., stimming) that supports Brent’s attention.

About half of the 80 partner turns in Video 3 (*n* = 39) had overlapping signing between Brent and his communication partner. Overlaps occurred when one conversation partner initiated their turn before the other partner had finished signing. Twenty-three overlaps were initiated by Brent (58.9%), while sixteen overlaps were initiated by his conversational partner (41%).

In summary, Brent appeared visually connected with his communication partner for the duration of the interaction; however, he demonstrated explicit signaling of his comprehension (backchanneling nods) or attention (handwringing) in only one-third of the partner’s turns. In addition, overlaps were common during the conversation.

Brent showed metalinguistic awareness in several instances. During Videos 1 and 2, Brent demonstrated audience awareness by making a side comment directed to his social media audience:

_1stPers_INFORM_2ndPers_ = “I’m just letting you (the audience) know.”

In Video 3, Brent recognized a point of miscommunication and subsequently engaged in conversation repair:

Brent: BABY GIRL LAST NAME, BABY GIRL LAST NAME

    ____________wh

Partner: BABY GIRL LAST

Brent: YOU BORN LAST NAME, YOU BORN LAST NAME

Partner: M-A-I-D-E-N N-A-M-E (Tilts her head back, mouths Ohhh!!, realizing that Brent is asking her what her maiden name was; she then fingerspells her maiden name).

Brent also engaged in sarcasm. In response to his partner’s question, YOU THINK THEY EAT EVERYDAY? (“(what) do you think they [residents of the town] eat every day?”), Brent responded DOG FOOD (“dog food”), and both he and his partner burst out laughing.

Brent’s expressive language included patterns that may be considered neologisms—novel words or expressions invented by a speaker that are not part of the standard vocabulary of a given language (Prelock, 2017). For instance, Brent fingerspelled F-F-M as a shorthand for “favor for me.”

We now proceed to a description of Brent’s nonmanual expressions.

### 3.2. Linguistic Facial Expressions

#### 3.2.1. Questions

We documented 104 questions across the 590 utterances, of which 31 were Wh-questions, 64 were Yes–No (polar) questions, 5 were rhetorical questions, and 4 were implied “which” questions by offering a choice (like “this or that?”).

Wh-Questions. Brent’s Wh-questions included a broad range of lexical signs, including WHAT, WHEN, WHERE, WHICH, WHY, HOW-MANY, HOW-OLD, and HOW. Twenty-two questions had explicit manual Wh-signs and nine had no explicit manual Wh-signs. Interestingly, in no case did Brent produce the expected furrowed eyebrows facial expression when signing a Wh-question. In one instance, Brent produced a chin raising movement as a possible alternate Wh-question marker while signing WHEN YOUR SPRING BREAK (“When is your Spring break”); however, this chin movement could serve an emphatic function as it occurred in what seemed to be a repeated phrase clarifying the first question (i.e., “When is spring break… no, I mean when is YOUR spring break?”).

Yes–No Questions. Brent produced 64 Yes–No questions across the five videos. Of these, all but three (95.3%) omitted the raised eyebrows facial expression; the three being produced with significant effort (with clenched mouth and slow, effortful raising of his eyebrows as if physically stretching).

Without raised eyebrows, it could be difficult for a signer to distinguish a Yes–No question from a declarative utterance, since the syntactic structure in ASL is often identical (still, the declarative statement may include a distinguishing affirmative head nod as another cue). However, Brent appeared to use several alternative strategies for marking Yes–No questions. First, he frequently added an explicit lexical “question mark” sign to his utterance, LEX-Q (*n* = 51, 79.7%). Second, he sometimes tilted his head (*n* = 9, 14%). Finally, we found one Yes–No question expressed without either a facial expression or one of these alternate markers (Video 3: HAVE RESTAURANT, meaning “does the town have a restaurant”). In this instance, the partner inferred from the context that this was a Yes–No question (based on the fact that she answered: HMM, NO). We note that Brent uses the LEX-Q strategy only with Yes–No (and not Wh-) questions, in accordance with the expected use of LEX-Q in ASL.

Rhetorical Questions. Brent asked five rhetorical-type questions across the videos; however, none of these were accompanied by the expected nonmanual marker (raised brow or head tilt) or an alternative strategy. For example, he asked:

___Rh

WHY C-O-V-I-D = “Why? Because of COVID” (Video 1)

 
in explanation of why he should not drink caffeine late in the day he asked:

___Rh

WHY KEEP-AWAKE = “Why? Because it’ll keep me awake” (Video 2)

Choice Offering. In four instances (Videos 3, 4), Brent used a “choice offering” syntactic structure to implicitly ask “which.” For example, he signed SMALL, MEDIUM, LARGE to ask “which size is it?”. In another instance, he signed YOU MEAN ASL O-R NICE ASL, which, in context, was interpreted to mean “which kind of ASL teacher are you, mean or nice?”. In each of these examples, Brent did not produce an accompanying Wh- facial expression, instead adding a body shift, bringing focus to each of the choices offered. Table 4 summarizes the results of our question marking analysis.

#### 3.2.2. Negation

We documented 26 expressions of negation across the five videos. Brent produced a headshake along with a manual negative sign in 21 (80.8%) of these utterances. For example, Brent signed the explicit negation signs NONE, WON’T, CAN’T with a headshake (*n* = 11). He also shook his head with signs that are manually modified to mark negation (e.g., DON’T-LIKE) (*n* = 4). He also added a headshake as a negator to non-negative signs, demonstrating the ability to use a headshake with a syntactic function (*n* = 6):

___________neg

IN NEWYORK = “not in New York”

and

____neg

MANY = “not many”)

In 5 of the 26 negation expressions, Brent signed a negative sign without the expected headshake (e.g., Video 2: NEWYORK NONE, with the interpreted meaning “there’s none in New York” and in Video 4: NOT VACCINE, meaning “those who weren’t vaccinated”). We noted that in 2 of the 5 omissions, Brent was using the choice offering type of question, with body shift nonmanual markers; following Gonzalez et al. (2019), the body shift may have taken priority over the negation headshake.

### 3.3. Affective Facial Expressions

Brent produced 50 affective facial expressions across the five videos. We noted Brent’s general disposition of a resting “grimace”^6^ so we were careful to not over-attribute negative affect. Of the 50 instances, 23 (46%) were expressed with a lexical sign with co-occurring (matching) facial expression, 22 (44%) were represented through facial expression only, and 5 (10%) were affective signs, but Brent produced them with a distinctive facial expression.^7^

Both positive and negative affect expressions were documented across the videos. While we did not observe other primary affective expressions such as fear or surprise, their omission in Brent’s repertoire may be simply explained by the context of his narratives or conversation not eliciting these emotions. We next report the affective expressions observed:

**Positive Affect**
Smiling (intermittently present in all videos);
HAPPY; TEASING; CALM; THUMBS UP; CELEBRATE (arms up waving)
#HAHAHA (# denotes lexicalized fingerspelling)
**Negative Affect**
Embarrassment: EMBARRASS (when mentioning a sign with a “naughty” implication)
Sadness: DISAPPOINT, SAD, CRY (when describing how he feels about a favorite store closing down); DEPRESSED; SUFFER
Hate/Dislike: HATE (when asserting that Mom hates a certain kind of pizza)

For the most part, when Brent conveyed positive affect and embarrassment affect, his facial expressions were consistent with expected ASL. However, sadness (*n* = 2) and hate/dislike (*n* = 3) expressions were somewhat distinct. When signing SAD, Brent’s mouth was open with seemingly heavier breathing, rather than the expected pout or downturned mouth. SUFFER also had a similar open mouth expression, with Brent also acting out the suffering, grabbing his shoulder in pain. In Video 2, Brent signed HATE (*n* = 2) with a slight squint and slight open mouth rather than the expected scrunched up nose and scowling mouth.

In Video 5, Brent produced five affective signs with a neutral facial expression^8^ (DEPRESSED; HAPPY; and the listed sequence CRABBY SAD HAPPY CALM). Importantly, as list structures can include a head nod accompanying each item, this nonmanual marker may be superseding the affective facial expression. In Video 3, Brent smiled in reaction to 13 (16.25%) of his partner’s 80 turns, had neutral affect in 66 (82.5%), and looked away once (1.25%).

We noted several instances of possible alternate means of affect expression. First, it appears that Brent conveys affect intensity through body movement rather than facial expressions (e.g., his sign SUFFER was followed by enactment of arm pain; EMBARRASS was followed by enactment of “oops” with hands-on-face and open mouth). Second, Brent modified his signing rate to mark intensity (e.g., the signs DEPRESSED and CALM were both produced more slowly to indicate very depressed or very calm). Third, we observed that Brent sometimes fingerspelled #HAHAHA, essentially providing a manual expression of smiling or laughing.

In summary, Brent expressed a range of affective meanings through his lexical signs, (e.g., HAPPY, SAD, DEPRESSED); however, his affective facial expressions were inconsistent, reduced, or distinctive. In some instances, he appeared to be using alternative strategies to convey affective states, such as manual signs and embodiment.

### 3.4. Other Nonmanual Expressions

#### 3.4.1. Head Movements and Body Shifts

Brent accompanied his signing with other nonmanual actions (*n* = 97) to serve different linguistic functions. These included nodding his head for affirmation (*n* = 42; e.g., Video 3: THUMBS UP; GENIUS; Video 4: BETTER; Video 5: DEAF), emphasis (*n* = 38; e.g., Video 1: SPICY; DELICIOUS; Video 2: COLD; Video 3: LARGE), and when expressing a list sequence of signs (*n* = 6; e.g., Video 3: FIRST, SECOND, THIRD; Video 2: FRUITS, VEGETABLES). He also moved his head laterally while signing an uncertainty-related sign (*n* = 2; e.g., Video 3: SOMETHING; SO–SO). Brent expressed disjunctions (*n* = 9; e.g., Video 3: YOU ATTEND DEAF-SCHOOL O-R MAINSTREAM WHICH) with an appropriate body shift from facing one option to facing another.

#### 3.4.2. Other Motor Behaviors

We observed several instances of nonmanual actions or behaviors beyond the 97 we documented above what may be best analyzed as stimming, including handwringing, eye rubbing, and lip licking or tongue movement. Brent wrung his hands in 7 of the 22 instances where he was backchanneling (head nodding) and five additional times during Video 3. It is notable that all instances of handwringing in Video 3 occurred only during his partner’s turns; he did not interrupt his own utterances with any handwringing.

Lastly, we observed one behavior that could be considered manual but not linguistic. During Video 2, Brent paused his signing to rub his eye. He immediately informed his audience that the eye rubbing was OFF-THE-POINT; that is, he was explicitly saying that his eye rubbing was not a sign (i.e., part of his linguistic expression). In another instance, Brent rubbed his eye before beginning his utterance and we noted that the eye rub action was not repeated across his subsequent phrasal repetitions, again suggesting this manual action was distinguished from Brent’s linguistic expression. Other behaviors, too numerous to count reliably, might be considered “stimming” actions for Brent (e.g., lip licking and pant leg rubbing).

## 4. Discussion

In this study, we examined 590 utterances by a Deaf, autistic, natively exposed, and fluent ASL signer (“Brent”). We hypothesized several possible ways that autism might interact specifically with facial expressions used for linguistic and affective functions in ASL. Given the findings from previous studies showing that hearing and deaf autistic individuals can show reduced or absent facial expressiveness for emotions/affect, we considered several possible predictions for Brent as it relates to the production of facial expressions for linguistic and affective purposes in ASL. First, early sign experience (and mastery of the linguistic use of facial expression) could possibly mitigate the impact of autism on Brent’s affective expressions (H0); second, autism could have a global effect on all facial expressiveness (H1); and lastly, autism may only affect facial expressions connected to emotion understanding and prosody and, thus, linguistic facial expressions would be unaffected (H2).

Our results are most aligned with H1, the prediction that autism may have a global effect on nonmanual facial expressions. Nonetheless, the patterns are somewhat more nuanced across linguistic and affective (nonlinguistic) domains, including Brent’s use of alternative strategies to convey some of these meanings.

### 4.1. Linguistic Expressions

The absence of facial expressiveness was most pronounced in Brent’s linguistic expressions, such as question marking facial expressions being virtually absent, compared to affective facial expressions, which were mostly present (for the affective contexts expressed) but possibly reduced and distinctive. Negation nonmanual markers (i.e., headshake) were present, but inconsistently used.

With regard to question marking, Brent produced a range of question formats but did not produce the expected furrowed eyebrows for Wh-type questions or raised eyebrows for Yes–No type questions (with the exception of three very effortful brow raises produced with Yes–No questions). He did utilize Wh-lexical forms, and a range of them, to ask his questions (e.g., WHEN, WHERE, WHICH, WHY, HOW-MANY, HOW-OLD, HOW). Since he typically included Wh- lexical forms, Brent’s communication partner was able to deduce that he was asking a question, despite the lack of accompanying facial expression. Brent also used rhetorical questions and choice offering questions without nonmanual markers. By contrast, Yes–No questions that lack the accompanying facial expression can become ambiguous, as there is no clear cue to indicate that the utterance is a question. Brent was only able to produce appropriate raised eyebrows on Yes–No questions with intentional effort. According to his mother, the use of raised eyebrows in this context is highly deliberate and self-initiated, without prompting. She noted that this was a skill he specifically worked on during recent months with one-on-one ASL instruction and that he is now actively practicing.

Importantly, Brent displayed alternative strategies to convey linguistic information that may be difficult for him to express via facial expressions. These strategies seem to be drawn from existing structures in ASL but were deployed more generally in Brent’s signing, and in some cases more distinctively. Brent frequently leveraged an explicit LEX-Q sign in polar questions to possibly replace the expected Yes–No brow raise; this strategy enabled Brent to clearly mark these utterances as questions for his interlocutors. Brent also asked Yes–No questions with head tilts and occasionally added emphasis with head or body movements.

Brent had a range of negation expressions (e.g., NO, NOT, NOT-YET, CAN’T), most of which (80.7%) were accompanied by head shaking. One indicator of Brent’s fluency with ASL linguistic structures is that he could also use the negation marker (headshake) with a syntactic function (e.g., adding a headshake while signing MANY = “not many”). Almost 20% of Brent’s negation-marked phrases had no accompanying headshake (and no observed alternative strategy that we could discern); however, careful review of these instances (*n* = 5) showed that two of them, NONE and NEVER, were expressed within a listing structure and a disjunction structure, respectively. As such, it is possible that the syntactic context could be blocking the presence of negative head shaking in these cases (see Gonzalez et al., 2019 for discussion). In the other three cases (NOT, NONE, NONE) we are uncertain why these occurred without a headshake. As a nonmanual-dominant signed language (Zeshan, 2006), a headshake is expected for negation in ASL. Brent had a strong command of head shaking as a nonmanual marker of negation but was inconsistent in its application.

### 4.2. Affective Expressions

Brent’s affective facial expressions (*n* = 62) were mostly positive affect (smiling, laughing) but also included some instances of negative affect (sadness, anger). Even with his starting point of a resting “grimace” face, Brent’s positive affect facial expressions were mostly recognizably consistent with what would be expressed by an ASL signer; his negative affect expressions, however, are noticeably distinctive (e.g., open mouth instead of pout for SAD). In some instances, we noted that Brent used an alternative, more manual, means to express emotional intensity, such as sign modification (slowed articulation) and body enactment. Both of these alternative strategies are available in the ASL linguistic repertoire (adverbial construction and constructed action). Additionally, Brent often used the fingerspelled sign #HAHAHA when he laughed, seemingly to make it explicit to his interlocutor that he is indeed laughing. This “manual laugh” is not obligatory when a signer is laughing; our impression is that its use in ASL conveys a bit of snarkiness (like in English “Oh, ha-ha, very funny”). We wondered whether using ASL’s #HAHAHA sign across a broad range of positive contexts offered Brent a manual way to compensate for his reduced or distinctive facial expressiveness.

Thus, these cases where Brent used sign modification, constructed action, and “manual laughing” suggested that Brent was utilizing his strong signing fluency to his advantage by specifying affective information (usually reflected through facial expression) through an explicit manual strategy.

### 4.3. Other Nonmanual Expressions

Brent appeared to use body enactment and movements (body shifts, head tilts) as alternatives to facial expression. We recognize that Brent’s stimming (e.g., lip licking, handwringing, and possible torso body movements) may also serve a communicative function. For example, his lip licking could be an alternate strategy to display emphasis, which would conventionally be expressed with an eye squint. His body movements, in addition to possibly being a stim, could also be demonstrating emphasis. These mouth or body stims could also be expressing an emotion such as joy (e.g., delighted to be eating s’mores). Manual stims, such as handwringing, could be disruptive to the coherence of a signed narrative; however, Brent did not engage in handwringing during his own utterances, but only when paying attention to his communication partner. Thus, Brent’s handwringing may have communicated to his partner, like he did with backchanneling, that he was paying attention and could be utilized as a regulation tool to support his turn taking (like de Marchena et al., 2019a utilizing gestures for regulating turn taking). Brent may be utilizing his strengths of signing fluency and larger head/body movements (compared to eyebrow movements) to communicate. However, Brent’s emphasis was sometimes over-expressed; while signers may lean forward or use a single head nod to add emphasis, Brent did it in a seemingly more exaggerated fashion.

### 4.4. Theoretical Implications

Given this, it appears that despite lifelong, native exposure to ASL and the lived experience of being Deaf, Brent exhibited a notable lack of linguistic facial expressions for ASL linguistic structures where they are regularly characterized as expected or obligatory, especially for questions. Brent showed some affective facial expressions, especially smiling, but his facial configurations (including his “grimace”) were distinctive, and possibly reduced. Importantly, however, Brent employed alternative strategies for expressing the information that ASL signers typically convey through facial expression. For affective expressions, Brent used manual sign modifications and body enactment, and for linguistic structures, he used manual markers (such as LEX-Q) and other body enactments.

### 4.5. Possible Mechanisms

Given our findings (global impact), we now turn to a discussion of the mechanisms that could be underlying the patterns observed: alexithymia, difficulties with prosody, motor challenges, differences in eye gaze behavior, and anxiety or depression.

#### 4.5.1. Alexithymia Account

The focus of facial expression research with autistic individuals has been on affective facial expressions. Studies have found differences in autistic individuals’ affective expressions (Trevisan et al., 2018) and one hypothesis is that alexithymia (difficulty identifying and describing emotions), rather than autistic traits, explains the differences observed in affective facial expressions (Trevisan et al., 2016). The underlying causes of alexithymia are not fully understood, but environmental, cultural, and genetic/neurobiological factors have been posited as potential contributors (Hogeveen & Grafman, 2021).

In the one previous study of facial expressions produced by deaf children with autism (Denmark et al., 2019), deaf autistic signers were found to produce affective facial expressions that were rated as lower in quality than typical deaf signers, and autism severity was associated with decreased facial expressions. They concluded that “deaf signing children with ASD show impairments in emotional facial expression production because deficits in emotion processing and theory of mind are central to autism and disrupt this ability in signers, as they do for users of a spoken language.” (2019: 301). While Brent also showed reduced affective facial expressions, this case study complicates this account because linguistic facial expressions were also largely absent. Additionally, Brent showed some awareness of his emotions through his production of multiple positive and negative affective signs. In any case, the alexithymia hypothesis fails to account for Brent’s lack of linguistic facial expressions in his ASL.

#### 4.5.2. Prosodic Account

An account related to alexithymia is the idea that disruptions in prosodic expressiveness could be responsible for differences in facial expressions in signers. Some studies have found that alexithymia is related to reduced emotional expressiveness through facial expressions and prosody (Trevisan et al., 2016). Prosodic expressiveness in hearing speakers relates to the rate, volume, stress patterns, and intonation of spoken utterances, which have long been characterized as atypical in autism (McCann & Peppé, 2003; Paul et al., 2005; Shriberg et al., 2001; but see also Grice et al., 2023 suggesting more mixed results).

Prosody in signed languages is expressed not only through the rhythm of manual signing but with various nonmanual markers, and there is disagreement in the literature about the status of these facial expressions. Some scholars have argued that facial expressions in signed languages behave like prosody in spoken languages, for example, by spreading over constituent phrases rather than individual lexical items (e.g., Atkinson et al., 2004; Herrmann, 2013; Nespor & Sandler, 1999). Others (e.g., Emmorey, 2002; Liddell, 1978, 2003; Neidle, 2000; Pfau & Quer, 2010; Zeshan, 2006) have argued that certain facial expressions in signed languages (such as brow raise in Yes–No questions and headshake in negation) are obligatory to the grammar and, as such, are not prosodic in nature. Wilbur (2000) has argued for a layered view of nonmanual markers, with distinct linguistic and prosodic systems functioning simultaneously: phonological nonmanual markers, which are tightly tied to lexical items (such as brow raise/furrow on questions and headshake for negation), and prosodic nonmanual markers (e.g., head tilts for emphasis; head nods aligned to signal intonational phrase boundaries), which can spread across phrases and signal intonation, clausal boundaries, or stress.

In our data, brow raise/furrow (nearly universally absent) and headshake (near universally present) did not pattern in the same way, so any account that treats them as belonging to the same linguistic class is difficult to reconcile. Brent is a fluent signer who signs rapidly and skillfully. Apart from facial nonmanual markers, his ASL syntax was considered acceptable (though repetitive), and he was able to both express himself clearly as well as comprehend the fluent signing of others. Thus, it is puzzling why he struggled with this particular element, facial expressions (brow raise/furrow), especially if it is expected among signers. Brent did not show any other morphosyntactic challenges, and he was able to use other linguistic nonmanual markers, such as headshake, appropriately.

Interestingly, the autistic linguistic savant Christopher exhibited a similar pattern when he was briefly exposed to British Sign Language: he was able to use negative headshake but did not produce linguistic facial expressions for marking questions (Morgan et al., 2002). That we find a similar pattern in an American, Deaf autistic native signer some twenty years later is remarkable.

All things considered, barring a motoric explanation for Brent’s specific difficulty with moving his eyebrows, the results are most parsimoniously explained if nonmanual markers are considered prosodic rather than linguistic and obligatory. Challenges with prosody in autism are well attested in the literature on hearing, speaking autistic people, while challenges with morphosyntax are less common (but see Eigsti et al., 2007 for some examples). If Brent’s issue was morphosyntactic in nature, we believe it would have appeared elsewhere in his signing.

#### 4.5.3. Motoric Account

Although motor challenges are not currently part of the formal diagnostic criteria for autism, fine and gross motor difficulties are common (McPhillips et al., 2014). It is possible that Brent’s overall reduced facial expressiveness (especially when it comes to moving his eyebrows) could have a motoric source. This interpretation would help to explain why we saw evidence of difficulty with both affective (nonlinguistic) and linguistic facial expressions (i.e., a global effect) rather than a selective difficulty in one area. Brent’s co-occurring Temple Syndrome could also be a factor, since it is characterized by differences in facial features, which could contribute to differences in facial expressions. We did not evaluate Brent’s motor skills formally, but such motor difficulties could account for the lack of question marking facial expression (characterized by relatively small eyebrow movements) and the simultaneous presence of negation (characterized by headshake, with relatively larger muscle activation). Brent also demonstrated the ability to produce other larger movements, such as head nods and body tilts, but only succeeded in producing eyebrow raises effortfully and infrequently (3 of his 64 Yes–No questions). Interestingly, these three instances occurred in a row: as he started this sequence, he stopped signing, raised his eyebrows with extreme effort and then kept them raised for the subsequent three Yes–No questions in a row. Thus, even in these few instances, Brent did not show the simultaneous integration of facial expressions and manual signing that would be characteristic of typical fluent signing (Brentari & Crossley, 2002; Sandler & Lillo-Martin, 2006).

#### 4.5.4. Eye Gaze Account

Another possibility is that differences in eye gaze behavior could lead to difficulties with producing facial expressions. Eye tracking studies have shown that (hearing) autistic people show reduced attention to others’ faces, especially the eye region of the face (Jones & Klin, 2013). Eye contact during conversation is often reduced, and difficulties with interpreting the facial expressions of others are common (see Harms et al., 2010, for a meta-analysis). Eye contact is a social (and functional) norm during ASL conversations, and the ability to glean information from the eyes and faces of others is essential for communication.

There is currently no work investigating the eye gaze behavior of deaf individuals with autism, and we did not analyze Brent’s eye gaze behavior here. However, we raise the possibility because difficulties with looking at the faces of others could lead to challenges with imitating and ultimately spontaneously producing facial expressions, even if such expressions are expected among signers. Since Brent has specific difficulties with facial expressions involving the eyebrows, but not with the movement of his head (as in nodding or shaking his head), we concede the possibility that a specific aversion to making eye contact with others could play a role here. However, our impression was that Brent maintained mutual eye gaze during conversation, lending weak support for this account in this case.

#### 4.5.5. Anxiety or Depression Account

One symptom of depression is flat affect (Pogue-Geile & Harrow, 1984), as there is reduced facial muscle activity over the brows and cheeks in depressed people compared to nondepressed individuals (Gehricke & Shapiro, 2000). To our knowledge, Brent’s depression was in remission at the time of these video recordings and we have examples of Brent smiling, laughing, and teasing. Thus, we do not think depression explains his nonmanual expression pattern. Anxiety, on the other hand, is associated with increased facial movements, particularly a fear expression. There is a high co-occurrence of anxiety in autistic individuals (between 11% and 84% of children; White et al., 2009) and like other autistic individuals, Brent might have been utilizing stimming to regulate his anxiety (Kapp et al., 2019).

Despite his anxiety, which might explain his stimming and sentence repetition, Brent displayed a reduction in facial expressions, contrary to what is typically observed in individuals with anxiety. Thus, it seems unlikely that anxiety explains his distinct pattern of nonmanual expressions.

#### 4.5.6. Summary

Thus, in light of the patterns observed, we find little evidence for the alexithymia and depression/anxiety accounts. We are somewhat dubious of the prosodic account (at least in its pure form), since Brent also showed difficulties with linguistic (non-prosodic) facial expressions. We did not measure Brent’s eye gaze behavior, but anecdotally we found that he made eye contact with interlocutors and did not avoid face gaze. Thus, we feel that a motoric explanation implicating specific facial muscles such as those responsible for moving the eyebrows is—at least for this particular signer—the most parsimonious explanation.

### 4.6. Deficit-Based vs. Strength-Based Approaches

A deficit-focused response to Brent’s nonmanual expressions could include framing his differences as deficits that are important to change. Such an approach may involve work focused on helping Brent to move his eyebrows, which places the communication responsibility solely on Brent. In the broader autism field, there is a shift from deficit-based approaches to more strength-based approaches (Urbanowicz et al., 2019). Lerner et al. (2023) described neurodiversity-affirming interventions as a balance between recognizing and celebrating the unique profiles of individuals while not ignoring existing difficulties. Strength-based approaches include positive social engagement, learning, self-advocacy, and anxiety reduction (Murthi et al., 2023). Thus, our recommendations for supporting Brent are centered in a neurodiversity-affirming, strength-based perspective.

A strength-based approach would view Brent’s communicative abilities holistically and would de-emphasize differences from normative signing that do not affect overall communication effectiveness. For example, Brent already uses a manual question marking particle (LEX-Q) as a substitute for eyebrow movements; he could be encouraged to expand his use of alternate strategies for conveying questions such as consistently using head tilts for Yes–No questions in addition to LEX-Q. Additionally, engaging with new communication partners may help to foster the development of these alternate strategies. Once Brent is aware of his strategies, Brent may also self-advocate by explicitly sharing them with his communication partners to facilitate their comprehension. Brent might find it useful to become more aware of his language patterns and consider whether he wants to adjust his languaging for greater effectiveness. It is crucial that any decision to modify his behavior is made by Brent himself, reflecting his own agency, consent, and sense of what is meaningful or beneficial to him.

Importantly, communication is bidirectional and Brent’s communication partners can also share responsibility for effectively communicating. Communication partners may reconsider their interlocutor role to take on more of the communicative load by, for example, learning his languaging patterns (e.g., also utilizing LEX-Q). Conversation participants can advocate for the primary goal of the interaction, that is, social connection; it may require nonautistic partners to become more intentional in supporting the autistic partner. For example, communication partners should not immediately assume the meaning behind Brent’s facial expressions, particularly given his tendency to display a more negative “grimace-like” face. Instead, they should learn from other behaviors or signs Brent uses to convey his thoughts and emotions. By closely observing Brent’s language patterns, communication partners may be able to detect shifts in his emotional state, such as increased anxiety or depression, allowing them to offer more appropriate and timely support.

Additionally, communication partners can be more supportive if they are aware of and adhere to the concept of “crip time” (Samuels, 2017), which refers to the flexible, individualized approach to time often necessary for disabled individuals with varying needs. For example, when Brent repeats his phrases, as he frequently does, partners could respond by being patient and recognizing this additional time needed before responding. Professionals who work with deaf autistic individuals can take an affirmative approach of recognizing and supporting languaging patterns rather than pathologizing non-normative language. Future research should seek to identify how autistic people are communicating in their own ways without characterizing these communicative styles as necessarily deviant.

### 4.7. Limitations and Future Directions

As with any case study, the current study has inherent limitations that restrict the generalizability of its findings. To our knowledge, this is only the second scientific study to specifically investigate the facial expressions of deaf autistic signers. More research is needed to confirm whether the patterns observed in Brent’s nonmanual expressions are consistent across a larger group of deaf autistic individuals.

Caution is warranted in the interpretation of our findings due to Brent’s comorbid Temple Syndrome, which can cause minor facial dysmorphisms and hypotonia. It is possible that some of the differences observed could be due to Temple Syndrome, autism, or the specific combination of the two.

An additional note of caution stems from our coding of facial expressions. Although our coding system achieved high interrater reliability across all categories coded in Brent’s spontaneous videos, we did not employ the gold standard Facial Action Coding System (FACS; Ekman et al., 2002), which codes facial expressions with a high degree of specificity and rigor.

Our study focused on narratives (four videos) and one conversation. Future studies could use more experimental measures such as elicitation of facial expressions, imitation of facial expressions, and comprehension of facial expressions by deaf autistic people. Since our data were naturalistic, spontaneous samples, we did not systematically elicit facial expressions, and it is possible that there are other facial expressions that Brent simply did not produce because he had no occasion to produce them.

A final limitation is the absence of an alexithymia measure. Without assessing alexithymia, we cannot know for sure whether Brent experiences alexithymia. Future work might collect a measure of alexithymia to better understand its interaction with facial expressions.

## 5. Conclusions

This case study contributes to the limited existing literature on the nonmanual expressions of deaf autistic individuals. In particular, we describe a fluent and capable signer who manages to express himself quite well and compensates for difficulties with certain nonmanual markers through the use of alternative strategies. Our work suggests that autism affects both linguistic and affective facial expressions, lending support to accounts grounded in motor difficulties and possibly prosody. Critically, our work suggests that the alexithymia hypothesis, rooted in emotion understanding, cannot account for Brent’s observed difficulties with linguistic facial expressions. Our work complements previous studies of the facial expressions of hearing autistic people, but adds a crucial element which has not been empirically tested before since non-signers do not employ facial expressions for linguistic purposes.

Finally, our work emphasizes the importance of affirming diverse languaging, recognizing that adhering to inflexible normative expectations around language may underestimate neurodiverse individuals’ communicative competence and lead to further marginalization rather than strengthening their abilities. As Dr. Jon Henner, a deaf advocate who worked in disability linguistics, profoundly stated, “how you language is beautiful. Don’t let anyone tell you your languaging is wrong. Your languaging is the story of your life.”

## Figures and Tables

**Table 1:** Predicted patterns of nonmanual expressions across domains.

	Functions of Nonmanual Expressions			
		Affective/ Prosodic Expressions	Linguistic Facial Expressions	Other Nonmanual Expressions
**Hypotheses**	**(H0) No impact**	Present	Present	Present
	**(H1) Global reduction**	Absent or reduced	Absent or reduced	Absent or reduced
	**(H2) Affective/Prosodic domain selectively affected**	Absent or reduced	Present	Variable

**Table 2:** Overview of video recordings.

Video Number	Age at Recording (Year; Month)	Length of Recording (M:Sec)	Topic of Discussion
1	16;11	10:20	COVID-19 pandemic, restaurants, favorite foods, and stores
2	17;4	8:01	Favorite restaurants, meals, stores, hotels, weather, and holidays
3	18;3	9:29	Restaurants: questions related to communication partner’s childhood, family, and schooling
4	20;0	2:12	Feelings about tough times, questions to his social media audience about their feelings, pets, and where they live
5	20;5	5:37	Sequence of April Fool’s pranks where Brent expresses “falsehoods” as questions to his audience

**Table 3:** Summary of nonmanual expressions used by ASL signers included in this study.

	Affect	Question Marking	Negation	Other Nonmanual Expressions
**Examples**	Happiness (smiling) Sadness (frowning) Anger (scowling)	Wh- (furrowed brows) Choice offer (furrowed brows) Yes–No (raised brows) Rhetorical (raised brows)	Shaking head	Affirmation or Backchanneling (nodding) Uncertainty (lateral head movement) Emphasis (emphatic head nod) Stimming

**Table 4:** Question marking summary across the five videos (*n* = 104).

	Wh-Questions (*n* = 31)	Yes–No Questions (*n* = 64)	Rhetorical Questions (*n* = 5)	Choice Offering (*n* = 4)
**With facial expression**	0 (0%)	3 (4.7%)	0 (0%)	0 (0%)
**Without facial expression**	31 (100%)	61 (95.3%)	5 (100%)	4 (100%)

## Data Availability

The data that support the findings of this case study are not publicly available due to the sensitive nature of the participant’s information and the high risk of identification. The participant is a member of a small and recognizable community (Deaf individuals with autism), and even with de-identification, there remains a risk of deductive disclosure. In accordance with institutional guidelines for case studies and ethical considerations around participant privacy, data sharing is not permitted.

## Notes

1 We follow the convention of using a capitalized ‘D’ for those who identify as culturally and linguistically Deaf, but also recognize recent work by Pudans-Smith et al. (2019) challenging whether this distinction is fully inclusive, especially of those with disabilities. In this paper, we used capital ‘D’ when referring specifically to Brent, his family members, and conversational partners in this study (all of whom are known to be fully integrated in the Deaf community and fluent ASL signers). Following Lillo-Martin and Henner (2021), we use lowercase ‘d’ in all other references. We use the term “natively exposed” to mean that a person is exposed to ASL from birth within a family context.

2 Brent’s repetitions were not copying other’s language (echolalia) and they were not repetitions of “favored utterances,” rather they were unique strings/phrases that he cyclically repeated in their entirety. He did not repeat single sign utterances.

3 We also acknowledge the possibility of a fourth hypothesis: that linguistic facial expressions will be selectively affected while affective/prosodic facial expressions will be preserved. However, seeing as there is little evidence in the literature for a selective difficulty in autism with grammar but not prosody or emotion, we have not included this possibility as a hypothesis.

4 The primary communication partner in Video 3 is a Deaf woman with other Deaf family members who works at a school for the deaf. Subjectively, we can report that she is a fluent signer of ASL. The other communication partner, a Deaf man, only exchanged a few turns with Brent, but, subjectively, we observed that he is also fluent in ASL (and know that he works as an ASL instructor).

5 Glossing refers to the conventionalized practice of representing signed language utterances using written words from a spoken language (e.g., English words represent ASL signs) in order to represent the lexical content and linguistic features of the signed production (e.g., Johnston & Schembri, 2007; Valli et al., 2011). Sign glosses are typically written in SMALL CAPS to distinguish them from spoken words. We follow the glossing conventions described in Sandler and Lillo-Martin (2006).

6 The term “grimace” is used here as a formal autism trait descriptor following the research literature (e.g., Esposito & Paşca, 2009; Tan et al., 1997).

7 Brent also signed some adjectives that could be interpreted as emotion-laden (e.g., TOUGH, HARD, TIRED), but we did not include them in this analysis as it was difficult to obtain coding agreement on whether these signs would be reliably associated with a particular affective facial expression.

8 Across the videos, there were 9 additional instances that we interpreted as an affective context, but Brent’s facial expression was neutral. In 8 of these 9 instances, Brent was signing a kind of “farewell” expression with I-LOVE-YOU handshapes on both hands, arms raised and circling, but with a neutral facial expression. We decided not to count this I-LOVE-YOU expression as an affect sign per se; even so, we felt the greeting was cheery and that most signers would express this sign with an accompanying smile.

## Appendix A

**Table A1:** Coding Scheme.

Code	Description	Real Examples from Videos
**Question marking**		
WhQSign_FE	Signs manual Wh-type question sign with expected furrowed brow facial expression	None
WHQSign_NoFE	Signs manual Wh-type question sign without expected furrowed brow facial expression	WHO, WHERE, WHY, WHICH, WHEN, WHAT, HOW, HOW MANY, HOW-OLD, WHAT-DO
WhQSign_Head	Signs manual Wh-type question sign without expected furrowed brow facial expression, adds a head nod	Signs WHEN YOUR SPRING BREAK and nods his head
No_WhQSign_NoFE	Context indicates Wh-type question but there is no manual sign or furrowed brow facial expression	MIDDLE NAME, MIDDLE NAME
No_WhQSign_Head	Context indicates Wh-type question but there is no manual Wh- sign or furrowed brow facial expression, adds a head nod	SMALL TOWN NAME, SMALL TOWN NAME
YNQuestion_FE	Signs Yes–No type question with expected raised brows	YOU HAVE CHILDREN LEX-Q; HAVE CAT LEX-Q
YNQuestion_NoFE	Context indicates Yes–No type question but there is no raised brow facial expression	HAVE RESTAURANT, HAVE RESTAURANT
YNQuestion_LexQ	Context indicates Yes–No type question and he adds the lexical form of “question” called “LEX-Q”	STEVE BROTHER HAVE TWO GIRL, LEX-Q
YNQuestion_Head	Context indicates a Yes–No question, without expected facial expression, adds a head movement	LIKE FISH (with an accompanying head tilt forward)
Rh-Q_FE	Poses a rhetorical type question with facial expression	None
Rh-Q_NoFE	Poses a rhetorical type question without facial expression	MAYBE YOUR MOM LOOK-YOU-OVER THINK YOU CAN WITH HEARING, WHY, YOU CAN TALK
Choice_Offer	Context indicates an offering of “and/or” structure or offering several choices which implies a question (“Which of these?”), without expected furrowed brow facial expression	SMALL, MEDIUM, LARGE TEACH MEAN ASL O-R NICE ASL?
**Affective**		
Aff-FacExp-Only	Shows distinct facial expression, absence of manual affect sign	Laughing and smiling while signing TWO SISTER WITH NICOLE
Aff-Sign-FacExp	Signs an affective sign (i.e., emotion word) with appropriate accompanying facial expression	Signs HAPPY with accompanying smile facial expression
Aff-Sign-Neutral	Signs an affective sign (i.e., emotion word) but an absence of facial expression	Fingerspells L-O-V-E without changing facial expression
Aff-Sign-Distinct	Signs an affective sign (i.e., emotion word) but his expression is distinctive	Signs SAD with open mouth, but not downturned
**Negation**		
Head_Neg	Only shakes head, absence of a manual negation sign	Responding “no” by only shaking head
Head_NegSign	Shakes head with presence of a manual negation sign	Shakes head while signing CAN’T, WON’T, DON’T, NEVER, NOT, NOT-YET, NO, NONE
Head_PhonMod	Shakes head with a sign that has been phonologically modified with a negation marker	Shaking head while signing DISLIKE, DON’T-KNOW, DON’T-WANT
Head_OtherSign	Shakes head with a sign that is not semantically encoded for negation or affect	Shakes head while signing PIZZA, CITY-NAME, SHH, PRETEND
NegSign_NoHead	Signs a negation sign, absence of headshake	Signs NONE, NEVER, CAN’T without a headshake
**Other nonmanual actions (During Brent’s Turn)**		
Uncert_Head	Moves head laterally with a sign that signals uncertainty	Bobbles head laterally while signing SO-SO, SOMETHING
OR-structure	Uses body shifting when expressing an _ or _ structure	NICE TO BOBBY O-R MEAN TO BOBBY
Emphasis	Body or head movement appears to be adding emphasis	NEW YORK CITY LARGE TOWN MANY STORE (when he signs LARGE, he leans forward as a kind of emphasis)
Head nod-Affirming	Nods head while signing phrase, as an affirmation	FIRST, SECOND, THIRD (with one nod accompanying each “buoy” in the list) MIKE GENIUS (while nodding affirmatively the whole phrase)
Lip licking	Mouth-Tongue action	
Stimming	Engages in some kind of nonlinguistic, manual or nonmanual, action while signing, excluding lip licking	Eye rubbing Handwringing
**Reaction: Interactive (Video 3 only)**		
Backchanneling	Brent nods his head while his communication partner signs	
Handwringing	Wringing his hands while his partner signs	
Backch + Handwringing	Both backchanneling and handwringing	
Other	Reacting with another response (e.g., head shaking) while his partner is signing	None
Not-Interact	Brent is not looking at partner while she or he is signing	Partner signs BYE at the end of Video 3 but Brent is not looking at her
Brent_OffScreen	No footage of Brent because the camera is on partner, not on him	
**Reaction to Partner: Affective (Video 3 only)**		
Positive-Aff	Smiling	
Neutral-Aff	Neutral, or grimace-like, facial expression	
Other-Aff	Any other affective facial expression	Sad or angry facial expression
**Overlapping (Video 3 only)**		
Overlap-Brent	Brent begins signing before partner has completed her turn	
Overlap-Partner	Partner starts her turn before Brent has completed his signing	Note: signing OH-I-SEE is excluded as “overlap” as it is appropriate backchanneling
